# Bioefficacy of some plant derivatives that protect grain against the pulse beetle, Callosobruchus maculatus

**DOI:** 10.1673/1536-2442(2006)6[1:BOSPDT]2.0.CO;2

**Published:** 2006-04-17

**Authors:** A. Rahman, F. A. Talukder

**Affiliations:** 1 Department of Entomology, Bangladesh Agricultural University, Mymensingh 2202, Bangladesh; 2 Department of Crop Sciences, College of Agricultural and Marine Sciences, Sultan Qaboos University, PO Box 34, Al-Khod 123, Oman

**Keywords:** Plant/weed derivatives, crop protectant, biorational pest control

## Abstract

Experiments were conducted to study the bioefficacies of different plant/weed derivatives that affect the development of the pulse beetle, Callosobruchus maculates F. (Coleoptera: Bruchidae) fed on black gram, Vigna mungo, *seeds.* Plant extracts, powder, ash and oil from nishinda (Vitex negundo L.), eucalyptus (Eucalyptus globules Labill.), bankalmi (Ipomoea sepiaria K.), neem (Azadirachta indica L.), safflower (Carthamus tinctorius L.), sesame (Sesamum indicum L.) and bablah (Acacia arabica L.) were evaluated for their oviposition inhibition, surface protectant, residual toxicity and direct toxicity effects on C. maculates. The results showed that plant oils were effective in checking insect infestation. The least number of F_1_ adults emerged from black gram seeds treated with neem oil. The nishinda oil extract was the most toxic of three extracts tested (nishinda, eucalyptus and bankalmi). Bablah ash was the most effective compared to the powdered leaves of nishinda, eucalyptus and bankalmi. The powdered leaves and extracts of nishinda, eucalyptus and bankalmi, at a 3% mixture, provided good protection for black gram seeds by reducing insect oviposition, F_1_ adult emergence, and grain infestation rates. The oil treatment did not show adverse effects on germination capability of seeds, even after three months of treatment.

## Introduction

The pulse beetle, Callosobruchus maculatus Fab. (Coleoptera: Bruchidae), is a major pest of economically important leguminous grains, such as cowpeas, lentils, green gram, and black gram (Talukder and Howse 1994; Okonkwo and Okoye 1996; Mulatu and Gebremedhin 2000; Raja et al. 2000; Park et al. 2003). The larvae bore into the pulse grain which become unsuitable for human consumption, viability for replanting, or for the production of sprouts. They are important pests of pulse crops in Asia and Africa under storage conditions (Ogunwolu and Idowu 1994; Okonkwo and Okoye 1996; Mulatu and Gebremedhin 2000; Raja et al. 2000; Ajayi and Lale 2001; Tapondjou et al. 2002).

Serious problems of genetic resistance by insect species, pest resurgence, residual toxicity, photo toxicity, vertebrate toxicity, widespread environmental hazards and increasing costs of application of the presently used synthetic pesticides have directed the need for effective, biodegradable pesticides (Zettler and Cuperus 1990; Glenn et al. 1994; Ewete et al. 1996; Guedes et al. 1997; Talukder and Howse 2000; Elhag 2000). This awareness has created worldwide interest in the development of alternative strategies, including the re-examination of using plant derivatives against agriculturally important insect-pests. Plant-derived materials are more readily biodegradable. Some are less toxic to mammals, may be more selective in action, and may retard the development of resistance. Their main advantage is that they may be easily and cheaply produced by farmers and small-scale industries as crude, or partially purified extracts. In the last two decades, considerable efforts have been directed at screening plants in order to develop new botanical insecticides as alternatives to the existing insecticides. It was reported that when mixed with stored-grains, leaf, bark, seed powder, or oil extracts of plants reduce oviposition rate and suppress adult emergence of bruchids, and also reduced seed damage rate (Talukder and Howse 1994; Onu and Aliyu 1995; Shaaya et al. 1997; Keita et al. 2001; Tapondjou et al. 2002).

The present research was carried out to determine the oviposition inhibition, surface protectant, residual toxicity and direct-toxicity effects of some local plant/weed derivatives against the pulse beetle, C. maculates fed on black gram, Vigna mungo, seeds. Effects of treatments on the viability of black gram seeds was also determined.

## Materials and Methods

Experiments on the efficacy of plant extracts, powder and oil as oviposition inhibitors, surface protectants, residual toxicants and contact toxicants against C. maculatus were conducted in the laboratory of the Department of Entomology, Bangladesh Agricultural University. All insect cultures were maintained in a growth chamber in the laboratory at a temperature of 27 ± 2° C, 12: 12 L:D and with 70 ± 5 *%* RH during the experiments. All experiments were conducted in a growth chamber under the same conditions.

### Test plant materials

Fresh plant leaves of nishinda (Vitex negundo L.), eucalyptus (Eucalyptus globules Labill.) and bankalmi (Ipomoea sepiaria K.); seeds of neem (Azadirachta indica L.), safflower (Carthamus tinctorius L.) and sesame (Sesamum indicum L.) were collected from the Bangladesh Agricultural University campus and neighboring areas during the winter seasons (November - February), washed and air-dried in the shade. Dried leaves and seeds were then ground to powder using an electric grinder. The powder, extract and oil from nishinda, eucalyptus, bankalmi, neem, sesame, safflower and ash of bablah (Acacia arabica L.) wood were used in this experiment. All plant extracts and safflower oil were prepared in the laboratory as described below, except that pure neem and sesame oil were purchased.

### Test insects and maintenance

The pulse beetle, C. maculatus F. was used for the present experiments. A small population of C. maculatus beetles was obtained from an entomology laboratory stock. They were reared and bred under laboratory conditions, on diet of the seeds of black gram, Vigna mungo, inside a growth chamber at 27 ± 2°C, with L:D 12: 12 and 70 ± 5% RH.

Initially, 50 pairs of 1–2 day-old adults were placed in a jar containing black gram seeds. The jars were sealed and a maximum of 7 days were allowed for mating and oviposition. Then parent stocks were removed and black gram seeds containing eggs was transferred to fresh black gram seeds in the breeding jars that were covered with pieces of cloth fastened with rubber bands to prevent the contamination and escape of insects. The subsequent progenies of the beetles were used for all experiments.

### Sample preparation of test plants

Powder and dust preparations of leaves were made by separately grinding approximately 500g of leaves of nishinda, eucalyptus and bankalmi in an electric grinding machine. The resulting powder was passed through a 25-mesh sieve to obtain a fine dust.

Acetone extracts were prepared according to the method of Talukder and Howse (1993) with modifications. Ten grams of ground leaves of nishinda, eucalyptus and bankalmi were separately mixed with 50 ml acetone and stirred for 30 minutes using a magnetic stirrer and then left to stand for 24 hours. The mixture was then filtered through Whatman #1 paper, and the solids were stirred again for 15 minutes with 30 ml of acetone and filtered and the filtrates were combined. The solvent from the pooled filtered solution was evaporated in a water bath at 65°C. After complete evaporation of solvents, the final crude extracts were weighed (1.72 g nishinda, 1.84 g eucalyptus and 1.58 g bankalmi), and preserved in sealed bottles in a refrigerator at 5°C until used for insect bioassays.

To prepare the oil extract of safflower seeds, one kg of seeds was ground in blender and soaked overnight in water. The soaked seeds were boiled for 2 hours and cooled. Scums that formed over the liquid were collected carefully leaving the residue below. The collected scum was boiled for extraction of oils. The pure neem and sesame oil were purchased from the local market.

Bablah wood was burned in a clean oven to produce ash. After cooling, the ash was put in sealed jar to prevent the absorption of air moisture. A 600 μm-diameter sieve was used to obtain fine ash.

### Oviposition inhibition effects

Laboratory tests for oviposition inhibition effects were conducted according to the method of Talukder and Howse (1994) with some modifications. Whatman filter paper disks (80 mm in diameter) were soaked in a 2 or 3% solution of an extract and air-dried for an hour. The control filter papers were treated with acetone only. The treated and control filter paper discs were placed singly at the bottom of Petri dishes (90 mm diameter) and 5g of black gram seeds were placed on the papers. Five pairs (5 female and 5 male) of C. maculatus beetles were released in each Petri dish which was covered for the next 7 days allowing them to lay eggs.

The adults were then removed and the following data were recorded:

Total number of seeds in each Petri dish
Number of eggs per 50 seeds in each Petri dish
Number of F_1_ adults emerged in each Petri dish from day 27 – 42
42 days after the setup the following data were recorded:
Percentage of eggs hatching = (Total egg hatch/Total eggs in each Petri dish) × 100
Inhibition rates IR% = [(Cn Tn) / Cn] × 100 (Where Cn = Number of insects in control dish and Tn = Number of insects in treated dish)

### Surface protectant effects

The tests for surface protectant effects were conducted according to the method of Talukder and Howse (1994) with some modifications. Diluted oil of neem, sesame and safflower were separately mixed with black gram seed at the ratio of 2.5, 5.0, 7.5 and 10.0 ml/kg seed. The oils were diluted with petroleum ether. Conical flasks, containing 40 g of seeds and oil mixture were shaken manually until the seeds were uniformly coated. After shaking, seeds were taken out and air-dried for one hour to evaporate the petroleum ether. Ten grams of treated or control seeds were put into each plastic pot (3.5 cm height × 4 cm diameter). Five pair (5 female and 5 male) of pulse beetle were released in each pot which were closed with a lid for 7 days to allow them to oviposit. The adults were then removed from the pots and data were recorded as described in the previous section.

### Direct toxicity by dipping method

The leaf extracts of nishinda, eucalyptus and bankalmi were diluted with acetone to make 2, 4 and 6% solutions. Five pair of adult insects (2–3 day old) were placed at the center of a piece of filter paper and the paper was twisted to enclose them. They were dipped in diluted extract or control solution for 35 seconds. The insects were removed, air-dried and returned to Petri dishes containing 5 g of black gram seeds. Four replications were made for each dose. Mortality was observed 24,48 and 72 hours after treatment. Insects were examined daily and those that did not move or respond to gentle touch were considered dead. Insect mortality data were corrected by Abbott's formula (1925), transformed into arcsin 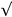
percentage values before ANOVA and then analyzed using ANOVA & Duncan's multiple range test (Duncan 1951). The concentration - mortality lines were calculated using probit analysis (Finney 1971) with a log10 transformation of concentrations of plant extracts. The results were expressed as concentration (%) per insect. Two LC50S were considered to be significantly different (P < 0.05) if their 95% fiducial limits did not overlap; slopes were similarly considered to be significantly different if their standard errors did not overlap.

### Residual toxicity test

A residual toxicity test was conducted according to the method of Talukder and Howse (1994) with some modifications. Ground leaf powders of nishinda, eucalyptus, bankalmi and bablah ash were mixed with black gram at the rate of 2% and 3% (w/w). The treated foods were then put into separate plastic pots (3.5 cm 4 cm), so that each pot contained 10 g of black gram seeds. Four replications were made of each dose. Five pair of adult beetles were introduced at the center of the pot containing the seeds and closed with a cover for 7 days to allow them to oviposit. The control pots contained untreated black gram seeds. The adults were then removed from the pots. The following observations were recorded:

Number of F_1_ adult emerging from each pot (from day 27 to day 42).
Seed damage rate from the random sample of 100 seeds at the end of the experiment,
Inhibition rates as described above
Percentage seed weight loss = [UNd DNu/U (Nd + Nu)] × 100 (where, U = Weight of undamaged grain, D = Weight of damaged grain, Nd = Number of damaged seeds and Nu = Number of undamaged seeds)

### Seed germination test

The viability of treated and control seeds were tested 3 months after the oil application. For this assay, black gram seeds were separately treated with the neem, safflower or sesame oil at the rate of 10 ml oil per kg seeds (1% v/w). The control seeds were treated with the solvent, but no oil or solvent was applied to the untreated seeds. The treated and control seeds were air-dried for 2–3 hours. Then 25 seeds from each treated, control or untreated group were placed separately in glass jars, under laboratory conditions but without insects, for 3 months. Each treatment was replicated four times. The germination of seed was evaluated for each treatment. Each group of seeds was placed on moist filter paper in Petri dishes. The dishes were kept in a incubator at 25°C and 12:12 L:D conditions. The dishes were observed for the germination of seeds for the next two months. Seed qualities were judged by tasting and smelling the treated seeds.

### Statistical analysis

Data were analyzed using a two factor completely randomized design using the different plant extracts and rates of application as the two factors. Mean values were adjusted by Duncan's Multiple Range test (Duncan 1951). For mortality tests, original data were corrected by Abbott's (1925) formula, transformed into arcsin & percentage values and then data were analyzed by probit analysis (Finney 1971).

## Results

### Oviposition inhibition effects of acetone extracts

The effects of different plant acetone extracts on oviposition are given in Table 1.

**Table 1.**
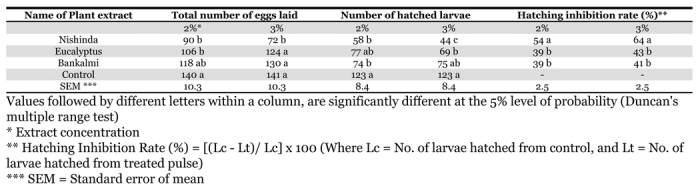
Effects of different acetone extracts on ovipostion of the pulse beetle, Callosobruchus maculatus.

The effects of different extracts on pulse beetles were evaluated by comparing the total number of eggs laid, egg hatching percentage and inhibition rates in the treated and control black gram seeds. The lowest number of eggs were laid in the food treated with nishinda, followed by eucalyptus and bankalmi at an extract concentration of 2%. The lowest hatching rate was found after treatment with bankalmi and nishinda leaf extract. Similar trends were noted for the 3% mixture. The highest oviposition inhibition rate was recorded in food treated with nishinda, followed by eucalyptus and bankalmi. Similar trends were noted for the 3% mixture.

### Surface protectant effect of different oils

The effects of treating the surface of the black gram seeds with oils of neem, safflower and sesame oil on C. maculates was investigated by comparing the number of F_1_ adult emerged, the total number of eggs laid, hatching percentage and inhibition rate using doses of 0.25, 0.50, 0.75 and 1.00% plant oils (Table 2). At a concentration of 0.25%, the lowest numbers of F_1_ adults emerged from seeds treated with neem oil, followed by safflower oil. The minimum numbers of total eggs were laid in the black gram seeds treated with 0.25% neem oil, and safflower oil. The lowest percentage of eggs hatching occurred from seeds treated with 0.25% sesame oil, followed by neem oil. The highest inhibition rate was counted from the seeds treated with 0.25% neem oil, followed by safflower oil. Higher concentrations of oil extract severely reduced emergence, hatching and oviposition.

**Table 2.**
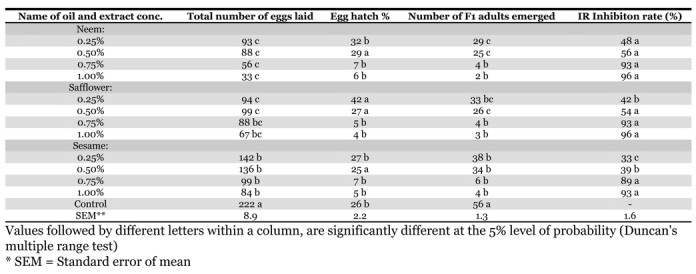
Surface protectant effect of different oils (v/w) on the pulse beetle, Callosobruchus maculatus

### Residual toxicity of powders and ash

The efficacy of different powders and bablah wood ash as grain protectants, was evaluated against C. maculates by comparing the number of emerged F_1_ progeny, seed damage rate, weight loss percentage and inhibition rates (Table 3). Female beetles were deterred from ovipositing in black gram seeds treated with leaf powders of nishinda, eucalyptus, bankalmi and bablah wood ash applied at the rate of 2% and 3% (w/w). At rates of 2 and 3 *%,* bablah wood ash and nishinda powder had similar effects on F_1_ progeny, seed damage, weight loss and inhibition rates.

**Table 3.**
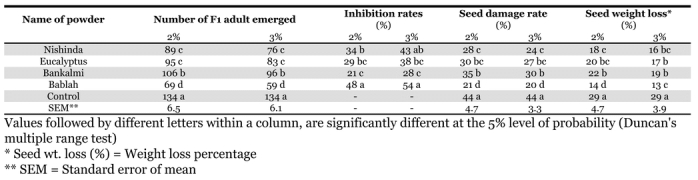
Residual toxicity of different powdered leaves and wood ash on the pulse beetle, Callosobruchus maculatus.

### Direct toxicity effects of acetone extracts

Insect mortality at 24, 48 and 72 hours after treatment, due to direct toxicity of acetone extracts of nishinda, eucalyptus and bankalmi leaves on C. maculates, was evaluated at three different rates 2, 4 and 6% (Table 4). The order of toxicity of the three extracts on pulse beetle was nishinda > eucalyptus > bankalmi. Mortality percentages were directly proportional to the extract concentrations and also with time after treatment.

**Table 4.**
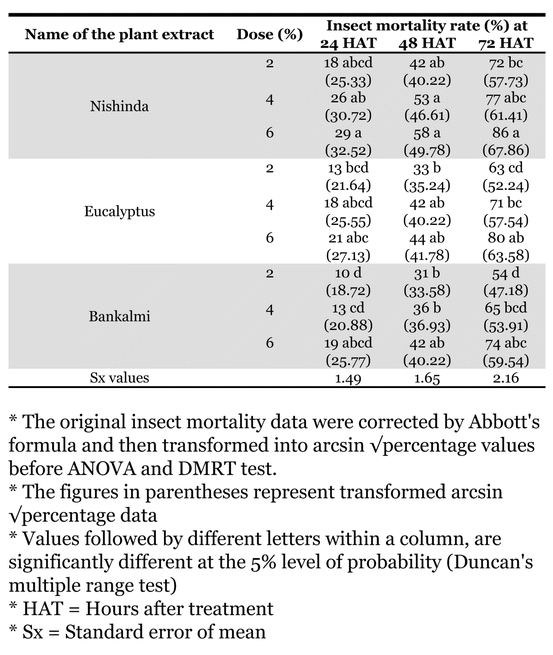
Direct toxicity effect (by dipping method) of different acetone extracts on pulse beetle, Callosobruchus maculatus F.

### Probit analysis of the effects of acetone extracts

The probit statistics, estimate of LC_50_ and their 95% fiducial limits and the slope of regression lines for 24, 48 and 72 hours after treatment are presented in Table 5. From probit analysis at 24, 48 and 72 hours after treatment it was found that the nishinda extract was the most toxic followed by bankalmi extract (Tables 5A–5C). The bankalmi extract had the lowest toxic on C. maculates. The extract of nishinda had the highest toxic effects against pulse beetle and lowest LC_50_ values. Higher concentrations contributed more significantly to the efficacy of extracts on the mortality of C. maculates and they appeared to be the most important factors in the degree of control obtained with different plant extracts.

**Table 5.**
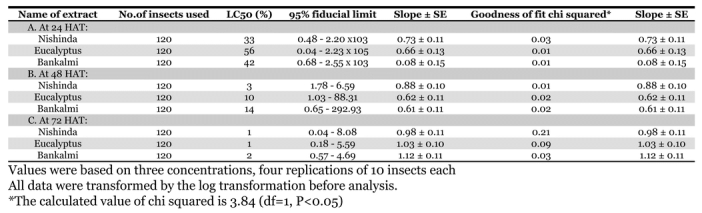
Probit analysis for direct toxicity at 24, 48 and 72 hours after dipping application of different acetone extracts to pulse beetles, Callosobruchus maculatus

When probit regression lines of the three different extracts were calculated, they showed a linear relationship between mortality percentage and extract concentration at 24, 48 and 72 hours after treatment. From the analysis, the regression line equations at 24 hours after treatment were Y = 3.895 + 0.731X for nishinda extract, Y = 3.688 + 0.662X for eucalyptus extract and Y = 3.251 + 1.079X for bankalmi extract. At 48 hours after treatment, the regression line equations were calculated as Y= 4.526 + 0.887X for nishinda extract, Y = 4.392 + 0.620X for eucalyptus extract and Y= 4.297 + 0.616X for bankalmi extract. At 72 HAT, the regression line equations were calculated as Y= 5.243 + 0.976X for nishinda extract, Y = 4.997 + 1.035X for eucalyptus extract and Y = 4.759 + 1.120X for bankalmi extract. Comparing all regression lines at 24, 48 and 72 hours after treatment, the regression lines for nishinda extract showed higher probit mortality in every case. The bankalmi treatment was more effective at providing immediate control of C. maculates, but its effectiveness decreased over time. In contrast, nishinda became more effective with increased time.

### Effect of different plant oils on seed germination

All the treated and control seeds were as viable as the untreated seeds (Table 6).

**Table 6.**
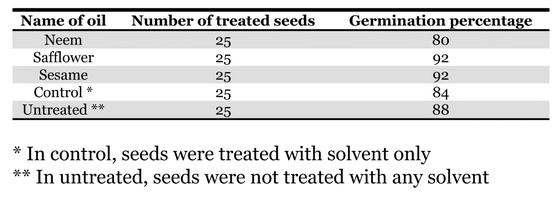
Effect of different plant oils (10 ml/kg) on black gram seed viability

## Discussion

### Effects of treated food on oviposition

Bhuiyan and Quiniones (1990) reported that nishinda leaf powder effectively prevented oviposition by the corn weevil. Talukder and Howse (1994) showed that the admixture of food with pithraj leaf, bark and seed powder reduced the oviposition rates of the pulse beetle. Srivastava *et al*. (1988) reported that eucalyptus oil effectively prevented the oviposition of insects. Mulatu and Gebremedhin (2000) showed that the oils of A. indica, Milletiaie ferruginea and Chrysanthemum cineraraefolium were the most effective in partially or completely preventing egg laying, and pulse beetles emergence from the laid eggs. Lale and Mustapha (2000) found no significant difference in the efficacy of neem seed oil and pirimiphos-methyl in reducing oviposition of C. maculatus, adult emergence or seed damage rates in treated cowpeas. Elhag (2000) tested extracts from nine plant materials as oviposition deterrents against C. maculatus and found that pulse treated with Rhazya stricta leaves, neem seeds, Heliotropium bacciferum aerial parts and citrus peels acted as highest ovipositional deterrents. Tapondjou *et al.* (2002) showed that the dry ground leaf of Chenopodium ambrosioides inhibited F_1_ progeny production and adult emergence of the C. chinensis and C. maculatus. These results are in general agreement with our findings.

### Effects of oil extracts

Oils of mustard, sunflower, safflower, castor and cotton acted as surface protectants against C. maculates population growth by reducing the seed damage rate and the number of F_1_ adults that emerged. Ketker (1989) observed that different oils of neem, coconut, and castor acted as surface protectants on green gram to check the pulse beetle and among them neem oil was the best surface protectant. Ramzan (1994) reported that cotton seed, sunflower, groundnut, soybean and mustard oils, when mixed with cowpea, completely suppressed adult emergence of C. maculates. Shaaya *et al.* (1997) reported that edible oils are potential control agents against C. maculatus and can play an important role in stored-grain protection. Ahmed *et al.* (1999) found that the neem and sesame oils completely inhibited adult emergence and appeared to be most promising as a seed protectant against C. chinensis. Yalamanchilli and Punukollu (2000) observed that the volatile oil from the leaves of Curcuma domestica could effectively protect the seeds, against C. chinensis, at a low concentration. In our present experiment, neem oil was the best protectant followed by the sesame oil.

### Residual toxicity

Mueke and Apuuli (1988) found that ash mixed with cowpea seeds gave satisfactory control of C. maculatus. Bioefficacy results from Lolage and Patil (1992) showed that neem, karanj, castor, groundnut and mustard oils significantly reduced seed damage rate from C. maculatus infestation. Seck (1994) reported that Securida longepedunculata leaf powder reduced or completely inhibited both emergence of F_1_ progeny of C. maculatus and seed damage. Ahmed *et al.* (1999) showed that after three days of release, 100% of the C. chinensis adults were found dead on neem oil-treated beans. Raja *et al.* (2000) reported that when jute bags treated with different plant leaf extracts including A. indica, V. negundo, C. collinus and J. curcas, and then used for cowpea seeds storage, the egg laying rates by the C. maculatus, adult emergence and seed damage were reduced. Raja *et al.* (2001) reported that when pulses were stored in gunny bags treated with aqueous extract from leaves of Melia azadirachta, Hyptis suaveolens and tubers of Cyperus rotundus, they effectively protected stored pulses without any infestation for up to 6 months. In contrast, our results show that bablah wood ash showed the most residual toxicity of the tested plant products.

### Direct toxicity

Bhaduri *et al.* (1985), found that the leaf extract of bankalmi had insecticidal properties against pulse beetle. Other researchers showed that different botanical extracts could be used for the control of pulse beetle. Ogunwolu and Idowu (1994) showed that 2.5% powdered seed of A. indica were toxic to C. maculatus. Mbata *et al*. (19951995) reported that dust and ether-extract from the seeds of the brown pepper were effective in enhancing the mortality of C. maculatus adults infesting cowpea seeds. Kim *et al.* (2003) showed the potent insecticidal activity of extract from cinnamon (Cinnamomum cassia) bark and oil, horseradish (Cocholeria aroracia) oil, and mustard (Brassica juncea) oil against C. chinensis, within 1 day after treatment. Okonkwo and Okoye (1996) reported that essential oils of Dennettia tripetela and brown pepper (Piper guineense) achieved 100% mortality of adults of C. maculatus in 24 h. Mulatu and Gebremedhin (2000) reported that eucalyptus seed powder treatment caused the death of emerging adult of Callosobruchus chinensis. In contrast to these studies, our results showed that nishinda acetone extracts were the most toxic.

### Effects of oils on seed germination

Das (1986), Onu & Aliyu (1995) and Keita *et al.* (2001) reported that seeds treated with botanical extract oils did not loose their viability. Onu and Aliyu (1995) reported that though various pepper powders were effective in reducing oviposition and damage of C. maculatus, seed quality and viability were not affected. Keita *et al.* (2001) reported that powders made from essential oils of different basils provided complete protection against C. maculatus, and also did not show significant effect on the seed germination rate. Our results confirmed these studies.

### Conclusions

The findings of the present investigations indicate that botanical derivatives might be useful as insect control agents for commercial use. All of the three powders and extracts tested were effective to some degree in reducing the ovipositional preferences and increasing the inhibition rates. Significantly fewer F_1_ adults emerged from food treated with extracts and powders. The highest F_1_ progeny inhibition was observed in black gram seeds treated with acetone extracts of nishinda leaves. Bablah ash provided better protection than three other powders. The neem oil was the most effective among three oils. To minimize the severe damage caused by insect pests, the traditional use of plant products, proved to be highly effective against stored-product insects. Application of plant/vegetable oils to grain seeds for storage is an inexpensive and effective technique, and its easy adaptability will give additional advantages leading to acceptances of this technology by farmers. A study to improve the effectiveness of botanical derivatives as insecticides will benefit agricultural sectors of developing countries, as these substance are not only of low cost, but also have less environmental impact in term of insecticidal hazard.
